# Prediction equations for maximal heart rate in obese and nonobese children and adolescents: a systematic review and meta-analysis

**DOI:** 10.1590/1984-0462/2023/41/2021397

**Published:** 2023-03-03

**Authors:** Maria Eduarda Casagrande Carli, Frederico Bento de Moraes, Francisco José de Menezes-Junior, Maiara Cristina Tadiotto, Jorge Mota, Neiva Leite

**Affiliations:** a Universidade Federal do Paraná, Curitiba, Paraná, Brazil.; b Universidade do Porto, Porto, Portugal.

**Keywords:** Heart rate determination, Pediatrics, Overweight, Exercise, Exercise test, Determinação da frequência cardíaca, Pediatria, Sobrepeso, Exercício, Teste de exercício

## Abstract

**Objective:**

The aim of this study was to analyze which equation best estimates maximal heart rate (HRmax) for the pediatric population according to body mass.

**Data source:**

We performed a meta-analysis (PROSPERO No. CRD42020190196) of cross-sectional studies that aimed to validate or develop HRmax equations and that had children and adolescents as samples. The search was conducted in Scopus, Science Direct, Web of Science, PubMed, and *Biblioteca Virtual em Saúde* with the descriptors “prediction or equation,” “maximal heart rate,” “maximum heart rate,” “determination of heart rate,” children, and adolescent. The TRIPOD Statement tool was used to assess the methodological quality and the relevant data were extracted for analysis. The meta-analysis was conducted in the Comprehensive Meta-Analysis, adopting p<0.05 and a 95% confidence interval (CI).

**Data synthesis:**

In total, 11 studies were selected, of which 3 developed predictive equations, 10 performed external validity of the preexisting models, and 1 incremented values related to equations already developed. The results of the methodological quality analysis showed a moderate rating in most studies. The 164 + (0.270 × HRres) – (0.155 × body mass) + (1.1 × METs) + (0.258 × body fat percent) (r=0.500, 95%CI 0.426–0.567, p<0.001) and 166.7+ (0.46 × HRres) + (1.16 × maturation) (r=0.540, 95%CI 0.313–0.708, p<0.001) equations presented stronger correlations with measured HRmax in nonobese adolescents. The predictive model developed by 208 – (0.7 × age) showed a greater accuracy among the possible models for analysis (SDM=-0.183, 95%CI -0.787 to -0.422, p=0.554). No specific predictive equation was found for obese adolescents.

**Conclusions:**

Future research should explore new possibilities for developing predictive equations for this population as a tool to control exercise intensity in the therapeutic management of childhood and adolescent obesity.

## Resumo

**Objetivo:**

Analisar qual equação melhor estima a frequência cardíaca máxima (FCmáx) na população pediátrica conforme a massa corporal.

**Fontes de dados:**

Foi realizada uma metanálise (PROSPERO n^o^ CRD42020190196) de estudos transversais que visavam validar ou desenvolver equações da FCmáx para crianças e adolescentes. As bases de dados foram Scopus, Science Direct, Web of Science, PubMed e Biblioteca Virtual em Saúde. Utilizaram-se os descritores “prediction or equation”, “maximal heart rate”, “maximum heart rate”, “determination of heart rate”, “children” e “adolescents”. A ferramenta TRIPOD Statement foi utilizada para avaliar a qualidade metodológica e os dados relevantes foram extraídos para análise. A metanálise foi conduzida no Comprehensive Meta-Analysis, adotando-se valor de p<0,05 e intervalo de confiança de 95%.

**Síntese dos dados:**

Foram selecionados 11 estudos, dos quais três desenvolveram equações preditivas, dez realizaram a validade externa de modelos preexistentes e um a incrementação de valores relacionados com equações já desenvolvidas. Em sua maioria, os estudos foram classificados com qualidade moderada. As equações 164 + (0.270 × FCrep) – (0.155 × massa corporal) + (1.1 × METs) + (0.258 × percentual de gordura) (2017) (r=0,500; p<0,001) e 166.7+ (0.46 × FCrep + (1.16 × maturação) (r=0,540; p<0,001) apresentaram correlações mais fortes com a FCmáx medida em adolescentes não obesos. O modelo de 208 – (0.7 × idade) mostrou a maior precisão entre os modelos possíveis para análise (SDM=-0,183; p=0,554). Não foi encontrada nenhuma equação preditiva específica para adolescentes obesos.

**Conclusões:**

Pesquisas futuras devem explorar novas possibilidades de desenvolvimento de equações preditivas para essa população, uma vez que elas são uma ferramenta para controlar a intensidade do exercício na gestão terapêutica da obesidade infantil e do adolescente.

## INTRODUCTION

Maximum heart rate (HRmax) is a parameter for intensity control of aerobic physical exercises, being part of the individual prescription for regular activities, therapeutic or cardiac rehabilitation programs, by using the HRmax percentual or reserve HR.^1,2^ HRmax can be directly measured using the maximum effort test;^3^ being defined as the highest HR reached, it remains on the plateau even with increased work intensity.^4^ It can also be predicted through equations,^5,6^ which are also used as a maximum effort criterion in the measurement of cardiorespiratory fitness (CRF).^6^

In daily practice and exercise programs, the HR range for training control is often calculated based on predictive HRmax equations, due to the cost and time available to perform the maximum test. Beyond that, overweight individuals have more difficulties generated by body fat overload to perform maximum efforts and reach the VO_2max_ plateau (CRF parameter), factors that interfere during the HRmax measurement.^7^ In addition, individuals with altered electrocardiogram, who have disabling comorbidities, and who need emergency equipment are not recommended to perform maximum effort,^1^ or even when the environment itself does not allow for the test to be performed.

However, the equations to predict HRmax have been developed using only age as a variable in their regression.^8^ The models developed by Fox et al.^5^ and Tanaka et al.,^6^ are most commonly used.^9^ Other predictive models were elaborated based on these two equations; however, the need to develop new ones for specific populations aiming lower prediction errors appeared.^10^ Since there are physiological differences between children, adolescents, and adults, such as lower stroke volume and higher HRmax in pediatric population,^11^ as a compensatory form for the smaller cardiac dimension, other variables, not just age, might influence HRmax prediction.^12–15^

In relation to obese population, there is still no consensus on which predictive equations are more appropriate. Miller et al.,^16^ developed a predictive equation for obese adults, claiming to have a lower predictive error compared to that developed by Fox et al.,^5^ showing an association between body composition and HRmax. However, Franckowaik et al.^17^ verified that this “new” model was overestimated compared to that of Tanaka et al..^6^ Therefore, HRmax predictive equations for the obese pediatric population have not yet been developed, which makes equations for nonobese subjects more widely used.

Considering the HRmax applicability and the difficulty that health professionals have in selecting the ideal predictive equation for a specific population, this study aims to answer the following question: “Which equation best estimates the HRmax for the pediatric population in relation to the body mass?” It was hypothesized that the models developed for the adult, youth, and physically active population would be inaccurate in predicting the HRmax of obese young people. Therefore, the purpose was to systematically review and perform a meta-analysis of evidence on the validity of different HRmax predictive models in obese and nonobese children and adolescents.

## METHOD

The search was carried out in August 2020, after registration on the basis of systematic review protocols (PROSPERO no CRD42020190196) and updated in February 2021, based on the recommendations of the Preferred Report Items Method for Systematic Reviews and Meta-analyses (PRISMA).^18^

The search was carried out in the Scopus, Science Direct, Web of Science, PubMed, and BVS (*Biblioteca Virtual em Saúde*) databases. The descriptors were selected based on the DeCS (*Descritores em Ciência da Saúde*)/Mesh (Medical Subject Headings), using the following terms in English: “prediction or equation,” “maximal heart rate,” “maximum heart rate,” “determination of heart rate,” children, and adolescent. The descriptors were combined with the Boolean terms “AND” and “OR”: (prediction OR equation) AND (“maximal heart rate” OR “maximum heart rate” OR “determination of heart rate”) AND (children OR adolescent). The search in the BVS database also used the same descriptors and combinations translated to Portuguese.

The following inclusion criteria were adopted:

- articles published until 2020;
- only original articles;
- cross-sectional studies;
- articles published in English, Portuguese, and Spanish; and
- studies with children and adolescents.

Exclusion criteria were as follows:

- studies not related to the theme;
- studies with animals;
- studies with a sample of adults only;
- studies with the elderly or individuals with respiratory and/or chronic diseases;
- measured HRmax through submaximal tests;
- intervention studies; and
- books, book chapters, monographs, dissertations, theses, review articles, case studies, abstracts, letters to the editor, editorial, and consensus.

The data were extracted into a spreadsheet previously elaborated with the following information: sample characteristics (mean age, mean HRmax, sex, and mean body mass index [BMI]), sample size, type of test (laboratory or field test), HRmax predictive equation and/or prediction equation developed in the study, and variables analyzed in relation to HRmax. The search was carried out by two authors (MECC and FBMJ), who independently reviewed potentially eligible titles and abstracts that met the eligibility criteria. Then, full-text articles were independently assessed. Disagreements were analyzed by a third author (MCT).

The selected articles were then examined for methodological quality using the TRIPOD Statement Scale,^19,20^ which consists of a checklist of 22 items, aiming to analyze the study report and assess the risk of bias and the clinical utility of developing, externally validating a prediction model, improving a prediction model, or even developing and performing external validation of the equation developed in the same study, whether for diagnostic or prognostic purposes.^19,20^ The results of the analysis were interpreted as low (≤50%), moderate (50–79%), or high (≥80%) methodological quality.

A meta-analysis was carried out with sufficiently homogeneous data in terms of statistical, clinical, and methodological characteristics, using Comprehensive Meta-Analysis. Values of sample size and correlation coefficients between the mean-measured HRmax and the predicted HRmax were obtained, and a significance level of p<0.05 and a 95% confidence interval (CI) were considered. In addition, the analysis of heterogeneity between studies was obtained from the I^2^ test, in which I^2^ of <25%, 25–50%, and >50% were considered small, medium, and large inconsistencies, respectively.^21^ The meta-analysis data were tabulated for better visualization.

The interpretation of the correlations performed in the studies were based on a single classification, in order to prevent different classifications between studies, as follows: very weak (0.0–0.19), weak (0.20–0.39), moderate (0.40–0.59), strong (0.60–0.79), and very strong (0.80–1.0).^22^

The sensitivity analysis was performed following the procedures:

- according to the type of stress test, field, or treadmill; and
- according to the test duration.

## RESULTS

**Figure 1.**
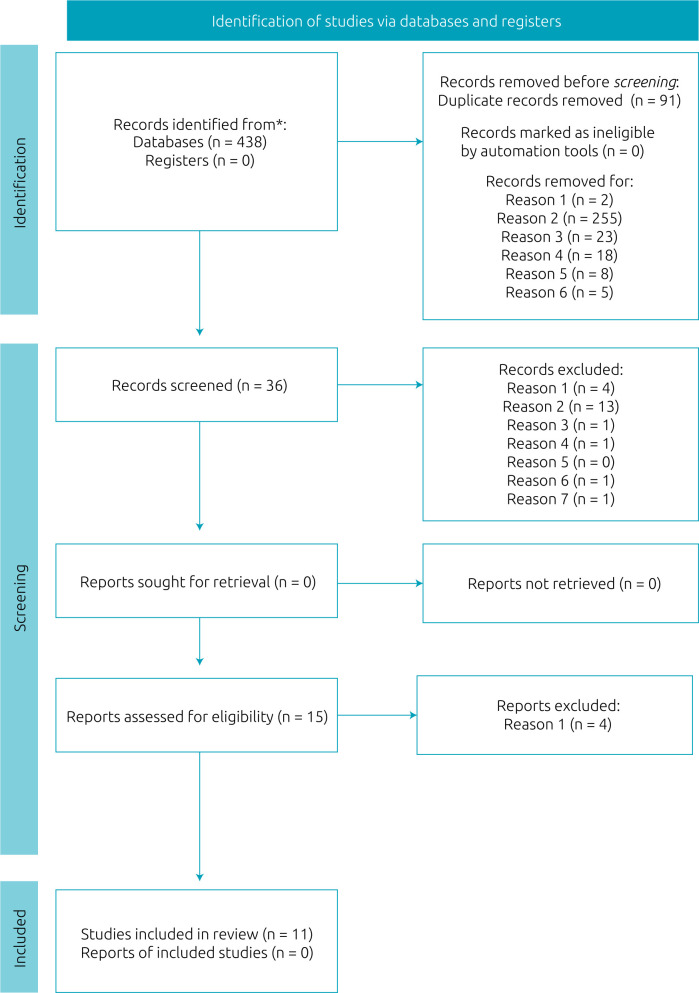
Systematic review flow chart detailing the identification, screening, eligibility, and inclusion of studies.

The search in the databases resulted in 438 records. After excluding 91 duplicates, 347 titles were analyzed, with 36 potential studies remaining for the analysis of abstracts. After screening, 15 studies were selected to assess for eligibility criteria. Therefore, 11 selected articles remained for the methodological analysis and data extraction. The selection of studies is shown in Figure 1.

Reason 1: studies that included only adults in the sample (n=10). Reason 2: studies not related to the theme (n=268). Reason 3: intervention studies (n=24). Reason 4: books, book chapters, monographs, dissertations, theses, review articles, case studies, abstracts, letters to the editor, editorial, and consensus (n=19). Reason 5: studies with measured HRmax through submaximal tests (n=8). Reason 6: studies with the elderly or individuals with respiratory and/or chronic diseases (n=6). Reason 7: studies without abstract (n=1).

The search resulted in three studies that developed new predictive equations: Mahon et al.^12^ classified with low methodological quality (50% score), Nikolaidis^23^ with moderate quality (68%), and Gelbart et al.^14^ with high quality (82%). Ten studies performed external validation, of which only one was evaluated with low methodological quality (50%),^24^ eight obtained scores between 50 and 70% attaining a moderate quality,^12–15,23,25–27^ and one scored 88%.^28^ A single study was carried out to increase values to preexisting equations^29^ and was classified as low quality (39%).

From 11 studies, 10 contained nonobese pediatric subjects^12–15,23–27,29^ and 1 contained obese pediatric subjects;^28^ of the 10 studies with nonobese sample, 7 included physically active young people.^14,15,23,24,26,27,29^ Regarding the criterion to consider the HRmax, 8 of the 11 included studies used the peak HR.^13–15,23,26,28,29^ Mahon et al.^12^ measured HRmax as the highest mean value obtained from two consecutive 15 s HR recordings. Also, two studies^24,25^ did not specify whether peak or plateau HR was measured.

Table 1 presents the study characteristics, as well as the summarized findings regarding predictive models. The equations that were validated externally by the studies are shown in Table 2.^4–6,10,12,16,30–38^ The model developed by Fox et al.,^5^ overestimated in most studies and the model developed by Tanaka et al.,^6^ diverged among the studies. Two studies developed new equations, i.e., one for children and adolescents in general^12^ and the other for athletes.^14^ The one for athletes had a lower standard error of estimate, with a low predictive capacity according to the authors.^14^ Relating to the variables that could influence HRmax, two studies did not find significant associations with age^12,13^ and one study did not find a significant correlation with gender and training level.^29^ HRres (15.6% contribution), body mass (5.7%), fat percentage (2.4%), and physical fitness level (1.2%) were identified as possible contributing factors for the prediction of HRmax and a significant correlation was observed with age (r=-0.278), height (r=-0.321), BM (r=-0.307), BMI (r=-0.190), and HRres (r=0.395).^14^

**Table 1.** Summary of selected studies.

Author	Sample characteristics		Study design		
	Sample age (mean±DP; years)	n (%female) BMI (kg/m^2^) and/or BMI-z (%)	Test protocol	Prediction equations	Overestimate, underestimate, or valid?
Mahon et al.^12^	Children and adolescents (12.00±3.10)	52 (40.38)	Incremental/treadmill	Fox Tanaka	Overestimate Valid
Machado and Denadai^13^	Adolescents (12.60±1.50)	69 (0.0)	Incremental/treadmill	Fox Tanaka	Overestimate Valid
Caputo et al.^25^	Adolescents (13.15±0.80)	23 (56.52)/20.80±2.55	Shuttle run	Fox Tanaka	Overestimate Overestimate
Colantonio and Kiss^29^	Untrained and trained adolescents (7–17 years; mean not mentioned)	145 (51.03)	Bruce/treadmill	Fox	Overestimate
Nikolaidis et al.^26^	Athletes (13.39±2.01)	47 (100%)/20.20±2.80	Shuttle run	Fox Tanaka	Overestimate Valid
Nikolaidis^23^	Athletes (15.80±1.50)	162 (0.0)	Modified Conconi	Fox Tanaka	Overestimate Underestimate
Souza et al.^24^	Athletes (16.89±1.28)	35 (not identified)	Incremental/treadmill	Fox Tanaka Nikolaidis	No associations found
Gelbart et al.^14^	Athletes (13.70±2.10)	433 (29.56)/19.90±3.40	Incremental/treadmill	15 equations	Underestimate higher measured HRmax. Overestimate lower measured HRmax
Cicone et al.^27^	Athletes (14.60±0.60)	30 (0.0)/20.30±2.10	Incremental/treadmill	Fox Tanaka Nikolaidis Shargal	Valid Underestimate Valid Underestimate
Heinzmann-Filho et al.^28^	Obese (16.80±1.20)	59 (56.0)/35.6±4.7/3.0±0.7	Adapted ramp/treadmill	Fox Tanaka Gellish Miller	Overestimate Overestimate Overestimate Valid
Papadopoulou et al.^15^	Active adolescents (13.30±0.70)	71(100.0)/21.10±2.2	Shuttle run	Fox Tanaka	Overestimate Underestimate

Test protocol description of the test protocol adopted in the study to measure HRmax; Prediction equation description of the equations that were evaluated in the studies; Overestimate, underestimate, or valid? the criterion adopted was from the results presented by the original studies; BMI body mass index; BMI-z body mass index score z; Fox: Fox et al.,^5^; Tanaka: Tanaka et al.,^5^; Nikolaidis: Nikolaidis^23^, Shargal: Shargal et al.,^30^; Gellish: Gellish et al.,^36^; Miller: Miller et al.^16^; HRmax: maximal heart rate.

**Table 2.** Predictive equations analyzed in the studies.

Studies	Subjects	Predictive equations
Edvardsen et al.^35^	Women	208−(0.66 × age)
Edvardsen et al.^35^	Men	220−(0.88 × age)
Fox et al.^5^	–	220−age
Gellish et al.^36^	General population	207−(0.7 × age)
Inbar et al.^33^	Men	205−(0.605 × age)
Itoh et al.^32^	General population	202.8−(0.763 × age)−(11.1 × sex)+(0.209 × (sex × age))
Londeree and Moeschberger^37^	General population	206.3–(0.711 × age)
Mahon et al.^12^	Children and adolescents	158.4+(0.44 × HRres)+0.68(age)
Miller et al.^16^	Obese adults	200−(0,48 × age)
Moss and Allen^34^	–	210−(0.65 × age)
Nes et al.^4^	Adults and elderly	211−(0.64 × age)
Nikolaidis^23^	Soccer players	223−(1.44 × age)
Robergs and Landwehr^10^	–	308.754 × (0.734 × age)
Shargal et al.^30^	–	201.104−(0.326 × age)
Tanaka et al.^6^	General population	208−(0,7 × age)
Whyte et al.^31^	Male athletes	202−(0.55 × age)
Whyte et al.^31^	Female athletes	216−(1.09 × age)

HRres: resting heart rate.

Mahon et al.^12^ equations:

Equation 1: HRmax=166.7+0.46(HRres)+1.16(maturation); R^2^=0.29; SEE=8.3; F(2)=9.96

Equation 2: HRmax=158.4+0.44(HRres)+0.68(age); R^2^=0.26; SEE=8.54; F(2)=8.54

Gelbart et al.^14^ equations:

Equation 1: HRmax=168+(0.259*HRres)-(0.156*BM (kg))+(0.891*METs)+(0.256*%FM) (R^2^=0.250, SEE=7.54 bpm)

Equation 2: HRmax=186+(0.25*HRres)-(0.14*BM) (R^2^=0.214, SEE=7.69 bpm)

In addition to these studies, another study developed a predictive model, obtaining SEE=8.6 bpm, with a moderate inverse correlation observed between HRmax and age.^23^

Nikolaidis^23^ equation:

HRmax=223-1.44×age (r=-0.27, SEE=7.6)

For obese adolescents, only one study was found^28^ which analyzed the equations developed by Fox et al.,^5^ Miller et al.,^16^ Tanaka et al.,^6^ and Gellish et al.,^36^ resulting only in the Miller et al.,^16^ predictive model as valid, while the others overestimating HRmax.

The meta-analyses were performed with five studies that analyzed the correlation between measured and predicted HRmax,^12–14,23,24^ including data from 751 children and adolescents between 10 and 19 years. Most of the equations showed a significantly weak correlation between the measured and predicted HRmax, thus a positive correlation between the variables. It was observed that the predicted HRmax using Fox et al.,^5^ (r=0.229; p<0.001), Tanaka et al.,^6^ (r=0.246; p<0.001), Nikolaidis^23^ (r=0.138; p<0.001), and equation 2 by Mahon et al.^12^ (r=0.354; p=0.001) (Table 3) are weakly correlated with the measured HRmax; all used only age as a variable of influence on the HRmax. The analyses were identified with high heterogeneity (I^2^=80.82%, p=0.005; 76.04%, p=0.015; 94.6%, p=0.000; 50.49%, p=0.155, respectively).

**Table 3.** Analysis of the correlation between HRmax predicted and measured by the equation developed by (a) Tanaka et al.^6^; (b) Fox et al.^5^; (c) Nikolaidis^23^; and (d) Mahon et al.^12^ and (e) by different studies.

Study name	Subgroup within study	Statistics for each study				
		Correlation	Lower limit	Upper limit	Z	p-value
a) Cross-validation Tanaka et al.^6^						
Gelbart et al.^14^		0.278	0.19	0.36	5.92	<0.001
Machado and Denadai^13^		-0.100	-0.32	0.14	-0.78	0.434
Souza et al.^24^		0.461	0.15	0.69	2.82	0.005
Overall effect		0.246	0.16	0.32	5.76	<0.001
b) Cross-validation Fox et al.^5^						
Gelbart et al.^14^		0.278	0.19	0.36	5.92	<0.001
Machado and Denadai^13^		-0.096	-0.32	0.14	-0.78	0.434
Souza et al.^24^		0.214	-0.13	0.51	1.23	0.219
Overall effect		0.229	0.15	0.31	5.37	<0.001
c) Cross-validation Nikolaidis^23^						
Gelbart et al.^14^		0.278	0.19	0.36	5.92	<0.001
Machado and Denadai^13^		-0.270	-0.41	-0.12	-3.49	<0.001
Souza et al.^24^		0.237	-0.10	0.53	1.37	0.172
Overall effect		0.138	0.06	0.21	3.47	0.001
d) Cross-validation Mahon et al.12 – Equation 2						
Gelbart et al.^14^		0.335	0.25	0.42	7.23	<0.001
Mahon et al.^12^		0.510	0.27	0.69	3.94	<0.001
Overall effect		0.354	0.27	0.43	8.11	<0.001
e) Cross-validation by different studies						
Gelbart et al.^14^						
CV Edvardsen et al.^35^		-0.023	-0.18	0.07	-0.48	0.633
CV Gellish et al.^36^		0.278	0.19	0.36	5.92	<0.001
CV Inbar et al.^33^		0.207	0.11	0.29	4.33	<0.001
CV Itoh et al.^32^		-0.030	-0.12	0.06	-0.62	0.534
CV Londeree and Moeschberger^37^		0.278	0.19	0.36	5.92	<0.001
CV Moss and Allen^34^		0.278	0.19	0.36	5.92	<0.001
CV Nes et al.^4^		0.278	0.19	0.36	5.92	<0.001
CV Robergs and Landwehr^10^		0.278	0.19	0.36	5.92	<0.001
CV Whyte et al.^31^		0.234	0.14	0.32	4.94	<0.001
CV Gelbart et al.^14^ – Eq. 1		0.500	0.43	0.57	11.39	<0.001
CV Gelbart et al.^14^ – Eq. 2		0.460	0.38	0.53	10.31	<0.001
Mahon et al.^12^						
CV Mahon et al.^12^ – Eq. 1		0.540	0.31	0.71	4.23	<0.001
Overall effect		0.258	0.23	0.28	18.28	<0.001

CV: cross-validation; Eq.: equation; In bold: statistically significant result (p≤0.05).

Moreover, the predicted HRmax by the two equations developed by Gelbart et al.^14^ (equation (1) r=0.500, p<0.001; equation (2) r=0.460, p<0.001) and one by Mahon et al.^12^ (equation (1) r=0.540, p<0.001) had a significant moderate correlation with the measured HRmax, which was expected since they were developed for children and adolescents. However, these studies showed high inconsistency (I^2^=92.1%, p<0.001). Still, these equations added other variables of influence on HRmax, such as body mass, HRres, %FM, METs, and maturation. It is worth mentioning that all equations explain less than 10% of the variations in HRmax.

The comparison results between measured and predicted HRmax (Table 4), with studies that presented sufficient data for analysis, showed that among the predictive models, the one developed by Tanaka et al.,^6^ underestimated, but not significantly, the measured HRmax, whereas Nikolaidis^38^ (p=0.008) and Shargal et al.^30^ (p<0.001) underestimated significantly. Moreover, Fox et al.,^5^ overestimated (p<0.001), as well as Gellish et al.^36^ (p<0.001) and Miller et al.,^16^ (p=0.031). All showed high inconsistency (I^2^>50%, p<0.001).

**Table 4.** Analysis of the difference between HRmax predicted and measured by the equation developed by (a) Tanaka et al.^6^; (b) Fox et al.^5^; and (c) by equation of several studies

Study name	Subgroup within study	Statistics for each study						
		SDM	SE	Variance	Lower limit	Upper limit	Z	p
a) Cross-validation Tanaka et al.^6^								
Mahon et al.^12^		-0.20	0.20	0.04	-0.59	0.19	-0.99	0.322
Machado and Denadai^13^		-0.20	0.17	0.03	-0.54	0.14	-1.15	0.249
Caputo et al.^25^		2.02	0.43	0.19	1.17	2.87	4.67	<0.001
Nikolaidis^23^		-0.71	0.13	0.02	-0.97	-0.45	-5.43	<0.001
Nikolaidis et al.^26^		-0.69	0.24	0.06	-1.16	-0.21	-2.85	0.004
Cicone et al.^27^		-2.08	0.51	0.26	-3.09	-1.08	-4.06	<0.001
Heinzmann-Filho et al.^28^		1.23	0.22	0.05	0.80	1.65	5.60	<0.001
Papadopoulou et al.^15^		-0.89	0.21	0.04	-1.30	-0.47	-4.22	<0.001
Overall effect		-0.18	0.31	0.10	-0.79	0.42	-0.59	0.554
b) Cross-validation Fox et al.^5^								
Mahon et al.^12^		1.20	0.23	0.05	0.75	1.66	5.20	<0.001
Machado and Denadai^13^		1.58	0.22	0.05	1.15	2.02	7.12	<0.001
Caputo et al.^25^		3.70	0.60	0.36	2.53	4.88	6.18	<0.001
Nikolaidis^23^		0.59	0.11	0.01	0.36	0.81	5.13	<0.001
Nikolaidis et al.^26^		1.40	0.26	0.07	0.90	1.91	5.46	0.004
Cicone et al.^27^		0.10	0.26	0.07	-0.40	0.60	0.40	0.689
Heinzmann-Filho et al.^28^		2.33	0.29	0.08	1.76	2.89	8.03	<0.001
Papadopoulou et al.^15^		1.40	0.21	0.04	0.99	1.80	6.68	<0.001
Overall effect		1.43	0.27	0.07	0.89	1.96	5.24	<0.001
c) Cross-validation by equations of different studies								
Cicone et al.^27^								
CV Nikolaidis^23^		-0.85	0.319	0.102	-1.472	-0.222	-2.657	0,008
CV Shargal et al.^30^		-2.52	0.597	0.357	-3.695	-1.354	-4.227	<0.001
Heinzmann-Filho et al.^28^								
CV Gellish et al.^36^		1.05	0.21	0.04	0.64	1.46	5.01	<0.001
CV Miller et al.^16^		0.40	0.18	0.03	0.04	0.76	2.15	0.031
Overall effect		-0.36	0.55	0.30	-1.43	0.72	-0.65	0.517

CV: cross-validation; SDM: standard difference in means; SE: standard error; In bold: statistically significant result (p≤0.05).

Sensitivity analysis for the protocols and duration of test cannot be generated as not enough data have been provided to perform it from the studies included in the meta-analysis.

## DISCUSSION

This study analyzed which HRmax equation for the pediatric population best estimated according to body mass, with the inclusion of obese subjects for analysis. Our results suggest that, in general, the equations developed by Gelbart et al.^14^ and Mahon et al.^12^ have a higher correlation with measured HRmax, and the model developed by Tanaka et al.,^6^ showed greater accuracy in estimating measured HRmax, as seen in other studies.^8^ It should be noted that we did not find enough data to analyze the difference between the measured and the predicted HRmax for the models developed by Gelbart et al.^14^ and Mahon et al.,^12^ which did not allow us to assess whether these models overestimate, are similar, or underestimate this variable.

For obese adolescents, only one study indicated that Miller et al.,^16^ model, which was developed for obese adults, presented less predictive error.^28^ However, in our study, this equation overestimated the measured HRmax. In addition, other models analyzed showed significant differences between the measured and predicted HRmax.^6,36^ Thus, it appears that HRmax predictive models have not yet been developed for obese children and adolescents, so the use of other equations could bring less accuracy to the estimation.

The inclusion of anthropometrics and body composition variables in the predicted models might bring more accurate predictions for obese and nonobese youth.^14^ When considering existing physiological differences between children/adolescents and adults, such as lower stroke volume and higher HRmax,^11^ only age does not seem to be sufficient to influence the prediction of HRmax;^6,30,36^ thus, authors indicate that there is no influence of this variable until puberty.^12–15^ An attenuated adrenal response of prepubertal adolescents in exercise when compared to postpubertal and adults, possibly due to sympathetic-adrenal regulation, is a possible influence over this variable.^39^

In addition, the selection of protocols, the duration of test, and ergometers can influence the development of a predictive method by interfering in the performance and consequently in the results of exercise tests.^40–42^ A limitation in this study was the lack of information to perform the sensitivity analyses for protocols and duration of test, but some points can be elucidated. The premise is that regardless of the protocol used, the tests must be maximal. However, field tests can be performed in small groups, which create a competitive environment that can influence greater effort on the part of the participants, besides not being monotonous. Corroborating this hypothesis, Berntsen et al.^41^ observed that the peak HR achieved during active play was higher than that achieved in treadmill tests in obese adolescents. Another study showed that high levels of perceived competence (intrinsic motivation) are associated with higher test performance.^43^

Regardless, some precautions should not be neglected, such as:

- the environment in field tests that cannot be controlled and can influence the HRmax by hot and humid conditions;^44^
- and the test duration that should range between 8 and 12 min to be considered adequate in relation to the work rate performed and not to fatigue-localized muscles.^42^

In relation to localized fatigue, we emphasized that the bicycle test essentially requires the strength of the thigh muscles.^45^ Therefore, the specific use of a muscle group may end up reflecting in a shorter test time due to localized muscle fatigue. One possible suggestion involving peak HR studies in juvenile population would be to adopt running protocols, seen as a fundamental human movement and to test the peak HR between field and treadmill protocols.

Our results show that the Tanaka et al.,^6^ equation would be the most suitable for use in children and adolescents, since it is the one that came closest to the measured HRmax among the models analyzed in our study. However, the applicability of predictive model developed by Tanaka et al.,^6^ in children and adolescents is still doubtful considering the noninclusion of individuals younger than 18 years old in its validation and cross-validation sample, but it is one of the most used equations in this population. This is a major limitation of studies with very wide age groups and that did not include categories of children and adolescents. Moreover, Nikolaidis^23^ found an estimated error of -3.2 bpm for adolescents and -5.0 bpm for adults with this model, an unexpected result, since the sample of the study by Tanaka et al.,^6^ was composed of adults and the elderly, thus expecting a smaller predictive error for this population.

Unlike the most used models,^5,6^ other predictive models developed added children and adolescents to their sample.^23,30^ However, both underestimated the measured HRmax in our meta-analysis, which was observed in another study that applied the same equations to a sample of young male soccer players.^27^ The smallest predictive errors in the study by Nikolaidis^23^ could be explained by the greater similarity between the samples involved in the study by Cicone et al.^27^ Moreover, the use of model developed by Tanaka et al.,^6^ showed greater SEE than that of Nikolaidis,^23^ which may suggest that the one specific for this population is more applicable, but it needs more studies for external validation of this equation.

According to sample characteristics, for nonobese children and adolescents, in general, the equations developed by Mahon et al.^12^ and Gelbart et al.^14^ seem to be more effective because they present greater correlations with the measured HRmax. The model developed by Gelbart et al.^14^ would be the most suitable for active nonobese children and adolescents, since their sample was composed of athletes, while Mahon et al.^12^ had active and nonactive participants, but it was not specified whether there were obese subjects.

The determination coefficient was higher for first equation developed by Mahon et al.,^12^ but the smallest predictive error was in the equation developed by Gelbart et al.^14^ with a greater number of variables, which may have influenced this result. However, the variables used in the models developed by Mahon et al.^12^ responded with less than 30% of the variance in the results and the standard error was not better than already observed in other equations, that is, the equations had low predictive capacity, but they were the ones that had the highest intensity in the correlations observed in our meta-analysis. It should be noted that Gelbart et al.^14^ indicated the use of 197 bpm as the average HRmax for children and adolescents, which has already been recommended by other authors.^13,14^

It is important to note that both studies had heterogeneous populations because they included nonpubertal and pubescent children, which can be a moderating factor in the development of the equations, since there are differences in the ages that girls and boys reach puberty.^39,46^ For future studies, we suggest to analyze puberty for possibly predictive models and to develop equations separately for prepubertal boys and girls. Besides, we noticed that there is a need for exploratory studies to identify anthropometric factors that consider the body surface of children and adolescents and are associated with HRmax in young people, such as BMI-z and triponderal mass index, which is efficient in predicting overweight in male adolescents^47^ and waist-to-height ratio.

Furthermore, fat accumulation may complicate locomotion in obese subjects and thus differ significantly from the HRmax achieved by their nonobese counterparts. When normalizing physiological values by body mass, large organisms may have lower values than small organisms.^48^ As a form of correction and comparison, the allometric exponent in body mass is used as a function to examine the relationship between the body surface and energy cost. In addition, mechanical efficiency and performance in weight-support sports are best determined by the allometric scale.^49^ Therefore, a large fat distribution may contribute to a worse performance in the maximal test, which ultimately influences test results such as HRmax. By adopting the allometric model that considers the effects of body size and body composition, the results can be better explained and thus are more accurate. With more studies in this area, we could promote a prediction equation more accurately and valid for this population.

The strength of this study resides in the gap found in relation to HRmax in this young, especially obese, population, and thus promotes guiding questions for future studies. This study had some limitations, such as the lack of data between studies selected to analyze the correlation and difference between measured and predicted HRmax, and the less number of studies that addressed the topic with obese adolescents, which did not allow us to have a valid conclusion for this population. Still, meta-analyses with a number of selected studies smaller than 20 end up having less power to detect heterogeneity.^50^

In conclusion, all equations were found to be unsatisfactory in predicting HRmax for obese and nonobese children and adolescents, with few validation studies and with high heterogeneity. In addition, studies that analyzed and included possible factors associated with HRmax besides age appear to improve the predictive models, but there is a necessity for more studies with a young sample. Thus, we suggest for future studies to analyze the pubertal stage and explore in further detail the relationship of anthropometric variables with HRmax, as well as the relationship between body fat distribution and maximal test performance, to increase the accuracy of predictive models. We reinforce the importance of the analysis of HRmax, so that there is greater effectiveness and control of physical exercise's intensity in their prescription for young individuals, since it is one of the therapeutic tools for the clinical management of children and adolescents with obesity.
